# Real-Time Genomics for Tracking Severe Acute Respiratory Syndrome Coronavirus 2 Border Incursions after Virus Elimination, New Zealand

**DOI:** 10.3201/eid2709.211097

**Published:** 2021-09

**Authors:** Jordan Douglas, Jemma L. Geoghegan, James Hadfield, Remco Bouckaert, Matthew Storey, Xiaoyun Ren, Joep de Ligt, Nigel French, David Welch

**Affiliations:** University of Auckland, Auckland, New Zealand (J. Douglas, R. Bouckaert, D. Welch);; University of Otago, Dunedin, New Zealand (J.L. Geoghegan);; Institute of Environmental Science and Research Limited, Porirua, New Zealand (J.L. Geoghegan, M. Storey, X. Ren, J. de Ligt);; Fred Hutchinson Cancer Research Center, Seattle, Washington, USA (J. Hadfield);; Massey University, Palmerston North, New Zealand (N. French);; Te Pūnaha Matatini: The Centre for Complex Systems and Networks, New Zealand (D. Welch)

**Keywords:** 2019 novel coronavirus disease, coronavirus disease, COVID-19, severe acute respiratory syndrome coronavirus 2, SARS-CoV-2, viruses, respiratory infections, zoonoses, coronavirus, epidemiology, genomics, New Zealand, community outbreak, border incursion, managed isolation, quarantine

## Abstract

Since severe acute respiratory syndrome coronavirus 2 was first eliminated in New Zealand in May 2020, a total of 13 known coronavirus disease (COVID-19) community outbreaks have occurred, 2 of which led health officials to issue stay-at-home orders. These outbreaks originated at the border via isolating returnees, airline workers, and cargo vessels. Because a public health system was informed by real-time viral genomic sequencing and complete genomes typically were available within 12 hours of community-based positive COVID-19 test results, every outbreak was well-contained. A total of 225 community cases resulted in 3 deaths. Real-time genomics were essential for establishing links between cases when epidemiologic data could not do so and for identifying when concurrent outbreaks had different origins.

Early in the coronavirus disease (COVID-19) pandemic, New Zealand (Aotearoa in Māori language) adopted a disease elimination approach. Elimination was first achieved in May 2020 (*1*,*2*), and through April 30, 2021, only 13 community outbreaks (Table) had occurred, comprising a total of 225 recorded community cases. We define a community case as illness in someone who has either been in contact with the wider community while potentially infectious or who was infected after being put into a dedicated managed isolation and quarantine (MIQ) facility because of one of the outbreaks discussed here. In contrast, we consider MIQ case-patients (i.e., returnees or MIQ workers) as those who acquired their infection through a chain of transmission that had not entered New Zealand.

**Table:** Summary of all coronavirus disease community cases in New Zealand since elimination was first achieved*

Outbreak	First case report date	Last case report date	Genomes	Community	Lineage(s)	No. unique genomes	No. segregating sites	Origin
Compassionate Exemption	2020 Jun 16	2020 Jun 16	2/2	2	A.11	1	0	MIQ
Auckland August 2020 Lockdown	2020 Aug 11	2020 Oct 20	155/179	179	C.12	40	44	Unknown
Rydges MIQ	2020 Aug 1	2020 Aug 16	2/2	1	B.1.509	1	0	MIQ
Crowne Plaza MIQ	2020 Aug 30	2020 Sep 22	9/9	6	B.1.36.18	4	4	MIQ
Sudima MIQ	2020 Oct 10	2020 Nov 3	24/33	2	B.1.1, B.1.1.397	1, 2, 7	0, 1, 14	MIQ
Sofrana Surville	2020 Oct 17	2020 Oct 22	3/4	4	B.1	1	0	Cargo vessel
Defence Force	2020 Nov 6	2020 Nov 18	5/8	6	B.1.36	1	0	MIQ
Airline Crew 1	2020 Dec 12	2020 Dec 12	1/1	1	B.1.1.7	1	0	Airline/ abroad
Pullman MIQ	2021 Jan 13	2021 Feb 4	3/5	4	B.1.351	2	1	MIQ
Auckland February 2021 Lockdown	2021 Feb 13	2021 Feb 28	14/15	15	B.1.1.7	4	3	Unknown
Airline Crew 2	2021 Mar 7	2021 Mar 7	1/1	1	B.1.1.317	1	0	Airline/ abroad
Grand Millennium MIQ	2021 Mar 13	2021 Apr 11	4/4	3	B.1.1.7	1	0	MIQ
Auckland Airport	2021 Apr 15	2021 Apr 20	2/2	1	B.1.1.7	1	0	Airport
Total	NA	NA	225/265	225	NA	NA	NA	NA
*Pangolin lineages are specified (version 2.3.9) (*3*) as well as the number of complete genomes/no. confirmed cases and no. community cases. No. unique genomes and no. segregating sites (i.e., no. genome positions that differ) within each cluster are counted, or within the 3 subclusters of the Sudima outbreak. All dates are the dates of report for the laboratory test. MIQ, managed isolation and quarantine; NA, not applicable.								

The public health response to community outbreaks differed according to the extent of the outbreak. Two outbreaks resulted in Auckland, New Zealand’s largest city, moving to alert level 3, which mandates stay-at-home-orders for most persons. The alert level system comprises levels 1–4; the most stringent is level 4 (*4*).

A core part of the COVID-19 elimination strategy is a strictly controlled border where nearly every person entering the country is required to isolate for 14 days at an MIQ facility and be tested for severe acute respiratory syndrome coronavirus 2 (SARS-CoV-2) on days 0, 3, and 12 of their stay (*4*,*5*). The MIQ facilities are repurposed hotels, more than half of which are located in Auckland. With an operational capacity of 4,000 returnees, the returnees (135,451 as of May 1, 2021) (*6*) and the considerable workforce required to service them present a possible transmission route into the community. Of the 13 known border incursions, 7 originated in MIQ facilities (4 from MIQ workers and 3 from returnees who tested positive after leaving the facility), 3 were from airline workers, and 1 was from an infection on a visiting ship; the sources of the remaining 2, which both led to stay-at-home orders, remain unknown.

Since April 19, 2021, travel between New Zealand and Australia has been open. Australia has also pursued an elimination strategy (although it is typically referred to as aggressive suppression) (*7*) and uses a hotel-based MIQ system although with notably fewer but larger outbreaks detected from their MIQ facilities (L.M. Grout et al., unpub. data, https://www.medrxiv.org/content/10.1101/2021.02.17.21251946v1) (*8*).

Viral genomic sequencing has played a crucial role in tracing and delineating all community outbreaks in New Zealand (*3*,*9*,*10*), complementing border controls, the alert level system, and contact tracing. There has been an effort to sequence the virus from every case-patient. Infected returnees in MIQ facilities are sequenced weekly, and community case-patients are sequenced more urgently; complete genomes are typically available to inform health officials within 12 hours of the first positive test result. Real-time genomic surveillance has been indispensable for confirming or disproving links between cases, particularly when epidemiologic data were lacking. We recount the events surrounding the 13 community outbreaks as of April 30, 2021, and demonstrate how genomic sequencing technologies have played vital roles in delineating these outbreaks.

## Materials and Methods

We constructed a multiple sequence alignment (*11*) containing 225 genomes from New Zealand community outbreaks and another 663 from the rest of the world, downloaded from GISAID (*12*). For each New Zealand outbreak, we sampled up to 50 global sequences from the same pangolin lineage(s) (*13*) as those of the outbreak, uniformly through time between the date of the first case in the outbreak and 60 days before. To reduce the effect of geographic sampling biases, global sequences were weighted proportionally to the number of sequences from the same country. For example, to sample the Pullman MIQ outbreak, we considered all B.1.351 global genomes collected up to 60 days before the outbreak and sampled 50 genomes from this pool, where the probability of sampling genome X was inversely proportional to the number of genomes in the pool from the same country as X. Our tree is the maximum-clade credibility tree summarizing a posterior distribution of trees inferred by BEAST 2 (*14*). Genomic sites were partitioned into the 3 codon positions, plus noncoding, as described (*3*)

For each partition, we modeled evolution with an HKY (Hasegawa-Kishino-Yano) substitution model with log-normal(μ = 1, σ = 1.25) prior on κ, frequencies estimated with Dirichlet (1,1,1,1) prior, and relative substitution rates with Dirichlet (1,1,1,1). We used a strict clock model with log-normal(μ = −7, σ = 1.25) prior on mean clock rate, and for the tree prior we used a Bayesian skyline model (*15*) with Markov chain distribution on population sizes and log-normal(μ = 0, σ = 2) on the first population size. We established convergence of the analysis by running multiple analyses (*8*) and using Tracer (*16*) to ensure that effective sample sizes were sufficient and that all individual analyses converged to the same distribution (supplemental information available at https://zenodo.org/record/5093838#.YOy_YDqxU5k). 

## Outbreaks

We reconstructed the phylogenetic tree for New Zealand’s border incursions by using complete viral genomes from New Zealand and, for context, from the rest of the world (*12*) (Figure; Appendix Figure). As of April 30, 2021, complete genomes (>90% recovery) have been obtained from 1,288 (57%) of 2,243 case-patients total and from 583 (57%) of 1,030 case-patients since June 1, 2020. For some case-patients without a full genome sequence, genomes were of lower quality, which was sufficient to assign them to a lineage, but for many case-patients, viral material was insufficient for any meaningful analysis. For the community outbreaks considered here, we had high-quality genomes for 225 (85%) of 265 case-patients (Table).

**Figure:**
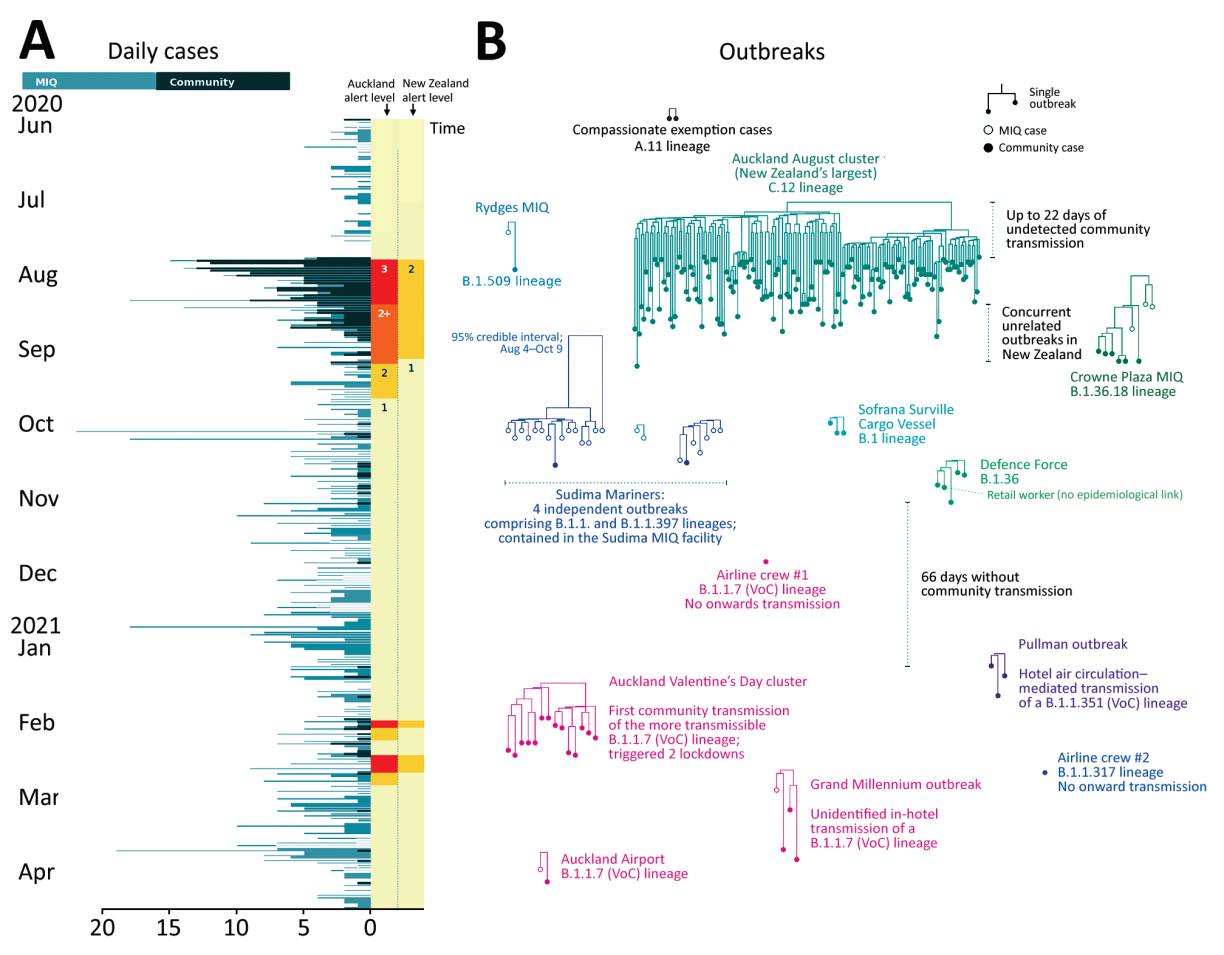
Outbreaks of coronavirus disease (COVID-19) after initial elimination in New Zealand. A) Daily COVID-19 cases, June 2020–April 2021. Alert levels in the Auckland region and across the wider country are indicated. B) Phylogenetic trees of all 13 COVID-19 postelimination community outbreaks. VoCs are indicated. Each subtree displayed is part of the larger phylogenetic tree built from genomes around the world. MIQ, managed isolation and quarantine; VoC, variant of concern.

### Compassionate Exemption

After 24 days without any recorded cases in the community or at the border and 1 week after all of New Zealand had been moved down to alert level 1, two cases were found in the community. These case-patients had arrived on June 7 and were granted a compassionate exemption to exit MIQ early to attend a funeral on June 13. The conditions of the exemption required them to self-isolate as much as possible while traveling and to get tested. They were both positive for COVID-19 on June 16. Within 1 day, complete viral genome sequencing confirmed that virus from the 2 case-patients shared a single origin. Although there were no secondary infections, concern for public health led to the New Zealand Defence Force being put in charge of managing MIQ facilities, no returnees being allowed to leave MIQ without having a negative test result, and ending compassionate exemptions from most of the MIQ requirements.

### Auckland August 2020 Lockdown

On August 11, 2020, a 102-day period with no recorded community transmission ended when 4 cases of COVID-19 were found among workers at an Auckland cold storage facility. The city was sent into an immediate lockdown (alert level 3), and lower level restrictions were introduced for the rest of the country (alert level 2). Elevated restrictions remained until October 7 as the cluster grew to a total of 179 cases, including 3 deaths. This COVID-19 cluster was the largest in New Zealand.

All genomes were closely related; 44 segregating sites were found across 155 genomes (Table). The single origin gave public health officials confidence that it was a single outbreak despite several cases having no clear epidemiologic links with other cases (*17*). The cluster included a healthcare worker who was infected during work at an MIQ facility where community case-patients were sent to quarantine (*18*). Although the index case-patients worked at a cold chain supply facility linked to the border, the source of the outbreak was never established (*19*). Complete genomes were available for 87% of cases in this outbreak, which we believe makes it one of the most comprehensively sampled large COVID-19 outbreaks.

### Rydges MIQ Facility

During the Auckland outbreak in August, there was an unusual case of COVID-19 in a maintenance worker at the Rydges MIQ facility with no known epidemiologic link to the ongoing community outbreak. Sequencing confirmed that the source of infection was not related to the main community cluster but rather to an overseas returnee under managed isolation at the Rydges MIQ facility. A follow-up investigation suggested that transmission probably occurred when the 2 case-patients used the same elevator minutes apart from each other (*20*) and that transmission was most likely airborne (*21*,*22*), although fomite transmission (e.g., through elevator buttons) cannot be ruled out.

### Crowne Plaza MIQ Facility

The complete Crowne Plaza outbreak has been thoroughly analyzed (*23*), so we provide only brief detail here. In September 2020, symptoms developed in a returnee who tested positive in Auckland 4 days after leaving the Crowne Plaza MIQ facility in Christchurch. Genomic sequencing showed that this case-patient (case-patient G) and 2 household contacts were not linked to the ongoing Auckland cluster in August. Rather, they were linked to other returnees under managed isolation at the Crowne Plaza. Epidemiologic investigations show that the chain probably started with case-patients A and B, who were seated close to case-patient C on a repatriation flight from India. Case-patient C then infected case-patient D in the MIQ facility via airborne transmission between a hotel room and its adjacent hallway. Case-patient D is then thought to have infected case-patient G on a domestic flight from Christchurch to Auckland. This outbreak illustrates the power of genome sequencing—when coupled with detailed epidemiologic investigations—for identifying cryptic transmission events such as those on flights or airborne transmission between MIQ guests. The genomic evidence here is complete; all sequences in the cluster are available, and the tree is relatively well-resolved, showing 4 distinct genomes among its 9 cases.

### Sudima MIQ Facility

On October 16, 2020, a group of 235 international mariners arrived in New Zealand on a charter flight from Moscow and began self-isolation at the Sudima MIQ facility in Christchurch. In the ensuing period, 31 of the mariners tested positive for COVID-19, as did 2 workers at the MIQ facility (*24*). Genomic sequencing produced full genomes for 24 case-patients and indicated >4 independent origins for the 33 cases; the viruses fell into 3 distinct phylogenetic clades (Figure). The 3 clades accounted for 2, 7, and 15 cases. We estimate the date of origin of the largest clade as between August 4 and October 9 (95% credible interval), but the mariners arrived on October 16, suggesting >2 separate introductions of this variant into the facility. The 2 MIQ workers were infected with 2 different variants of the virus; public health officials concluded that the 2 transmission events occurred via regular interactions with the mariners where protocols were followed but were insufficient to prevent transmission (*25*).

### Sofrana Surville

In mid-October, routine testing indicated that a border worker was positive for COVID-19. Extensive surveillance testing found 3 others who were positive: 2 were household contacts and 1 was a mariner who worked on the same cargo vessel, the Sofrana Surville (*26*). Genomic testing confirmed that virus isolates from the 4 case-patients shared the same origin and were part of a lineage that was novel to New Zealand, thus making residual community transmission an unlikely explanation. International crew members on the Sofrana Surville were confirmed as the source of infection when the ship arrived in Australia, and crew were tested by health officials in Queensland. One crew member tested positive, and the virus sequence reportedly matched that of the New Zealand case-patients.

### Defence Force

After visiting several public locations in the Auckland central business district, a New Zealand Defence Force member tested positive for COVID-19 (November 2020). Genomic sequencing confirmed that his infection was acquired from the quarantine facility where he worked. Contact tracing identified 3 additional cases in Wellington (500 km from Auckland, where the Defence Force member’s close contact resided), and surveillance testing identified 1 community case in the Auckland central business district. The only known connection to the rest of the outbreak was that this case-patient worked at a retail outlet ≈50 m from one of the locations visited by the index case-patient (*27*). The case was quickly linked to the rest of the cluster through whole-genome sequencing, providing reassurance that widespread undetected community transmission was unlikely (*28*). The circumstances of the transmission event remain unknown. Because the genomic link was established, the alert level was not changed. However, the general public was asked to avoid the Auckland central business district, if possible, for ≈3 days.

### Airline Crew 1

In December 2020, an airline crew member tested positive for COVID-19. The worker was self-isolating at an airline hotel-based facility (as opposed to a dedicated MIQ facility) because of return from a high-risk country (United States). The crew member tested positive within the first 48 hours of self-isolation, and there were no recorded secondary infections (*29*). Genomic sequencing indicated that the infection had been acquired abroad. Although this case meets our definition of a community case in that it occurred outside one of the MIQ facilities, the wider community was at little risk, and the case was managed according to aircrew protocols (*30*).

### Pullman MIQ Facility

A returnee (case-patient A) tested positive for COVID-19 1 week after completing managed isolation at the Pullman MIQ facility (January 2021). Despite extensive travel across the Northland region while infected with the B.1.351 lineage (Beta variant) of SARS-CoV-2 (H. Tegally et al., unpub. data, https://www.medrxiv.org/content/10.1101/2020.12.21.20248640v) (*31*), no secondary infections were reported, including in case-patient A’s traveling companion. Shortly thereafter, 2 additional cases (case-patients B and C) with the same variant (and their household contact) were found in the community; case-patients B and C had completed self-isolation at Pullman (*32*). The outbreak was successfully limited to these 4 community cases, which were genomically linked to a returnee under isolation at Pullman. Case-patient A had occupied a room on the same floor as the source case-patient and may have been infected through the air circulation system; whereas, case-patient B was most likely infected from using an elevator 3 minutes after the source case-patient, despite their use of face masks (*33*). New arrivals at the MIQ facility were suspended, and its air filtration systems were improved.

### Auckland February 2021 Lockdowns

On February 13, 2021, a total of 3 household members in Auckland tested positive for SARS-CoV-2. The genomes were identical and of the highly transmissible B.1.1.7 (Alpha) variant (*34*,*35*). Although 1 case-patient worked at an airline services company, that person had no obvious contact with persons coming through the border. Auckland was immediately sent into its third alert level 3 lockdown for 3 days, and widescale surveillance testing commenced. Other cases with closely related virus genomes were found; however, the epidemiologic links were not always strong (*36*). A fourth alert level 3 lockdown followed shortly after, when another case was found that could not immediately be linked to the cluster.

The known outbreak was restricted to 4 households and 15 cases, although epidemiologic gaps suggest that there may have been undetected cases. Genetic evidence showed 4 distinct genomes among 14 cases, which was not informative beyond confirming a single origin; the B.1.1.7 variant’s global overrepresentation presented further difficulties at pinpointing an overseas origin. The outbreak has not been traced back beyond the original 3 cases, and its origin remains unknown.

### Airline Crew 2

During the Auckland February outbreak, an airline crew member tested positive 1 week after arriving from Japan (*37*). Self-isolation was not required because the worker did not arrive from a high-risk location. There were no known secondary infections, including household contacts. Genomic sequencing suggested that the infection was most likely acquired overseas.

### Grand Millennium MIQ Facility

In late March 2021, a cleaner at the Grand Millennium MIQ facility tested positive. Despite this person being infected with the more transmissible B.1.1.7 lineage, there were no known secondary infections in the community. Two weeks later, 2 other workers from the same facility tested positive. All 3 cases were genomically linked back to a returnee isolating at the Grand Millennium.

### Auckland Airport

A worker at Auckland International Airport tested positive for the B.1.1.7 variant in April 2020. No onward transmission was detected. Genomic sequencing linked the case to a recent returnee, and a follow-up investigation showed that the infected worker had cleaned the airplane on which the returnee had arrived, so it was likely a case of fomite or airborne transmission, despite the worker wearing personal protective equipment (*38*).

## Discussion

Real-time genomic sequencing has been used to investigate each of the 13 COVID-19 community outbreaks after the initial elimination in New Zealand. Sequencing has been essential, not only for establishing links between cases when epidemiologic links could not (e.g., the Defence Force outbreak and both Auckland lockdowns) but also for identifying when multiple outbreaks had different origins (e.g., decoupling the Rydges outbreak from the ongoing Auckland outbreak and the second airline crew case from the February 2021 outbreak). These efforts have been instrumental in clearly delineating outbreaks and informing the public health response. Genomic sequencing has also elucidated cryptic modes of transmission, such as airborne transmission (*23*) and in-flight transmission (*39*), which have brought about policy changes (e.g., revision of filtration systems in MIQs).

When paired with routine genomic sequencing from within MIQ facilities and around the world, genomics can identify the origins of community outbreaks and rule out the possibility of undetected widespread community transmission. However, as exemplified by the 2 community outbreaks that led to lockdowns, the ability of this strategy to identify outbreak origins when there is no closely matched genome with a plausible epidemiologic link is limited (*18*).

A wide range of lineages have been imported into New Zealand over the course of the pandemic (Table) (*8*). The fact that the 4 outbreaks of locally acquired infection in 2021 were all caused by variants of concern (VoC)—pangolin lineages B.1.1.7 and B.1.351 (*13*,*40*) (P. Wang et al., unpub data, https://www.biorxiv.org/content/10.1101/2021.03.01.433466v1)—reflects the lineages that are arriving at the border. A total of 83 of the 142 genomes from overseas returnees found during January 1–April 30, 2021, were from those 2 lineages or other VoCs. However, data are too scarce to make any link between these outbreaks and the reported higher transmissibility of the VoCs.

New Zealand’s ability to rapidly generate SARS-CoV-2 genomes has greatly improved over the past year, to the point where new genomes are routinely available within hours of positive community test results. Although the potential of the techniques described here has been well characterized in academia (*41*), the pandemic has facilitated their widespread adoption in New Zealand and other places (e.g., Singapore and Australia) (*42*,*43*); the term “whole-genome sequencing” is becoming commonplace in public health announcements. Combined with epidemiologic investigation, those data have increased public knowledge of the outbreak and have driven policy change. Although the techniques described here of real-time sequencing and analysis coupled with epidemiologic investigation have come to the fore during the COVID-19 pandemic, they are not limited to pandemic situations. These technologies can be integrated into regular surveillance of other pathogens, such as seasonal influenza viruses, which have been largely absent from many countries over the past year (*44*).

Appendix Complete phylogenetic tree for severe acute respiratory syndrome coronavirus 2.
