# Supplementary material for: Real-Time Genomics for Tracking Severe Acute Respiratory Syndrome Coronavirus 2 Border Incursions after Virus Elimination, New Zealand
Source: Emerg Infect Dis. 2021 Sep;27(9):2361–8. doi: 10.3201/eid2709.211097 (PMC8386796; doi:10.3201/eid2709.211097)
Supplement: Appendix — Complete phylogenetic tree for severe acute respiratory syndrome coronavirus 2. [file 21-1097-Techapp-s1.pdf]

Article DOI: https://doi.org/10.3201/eid2709.211097

Real-time Genomics for Tracking Severe Acute Respiratory Syndrome Coronavirus 2 Border Incursions after Virus Elimination, New Zealand

Appendix

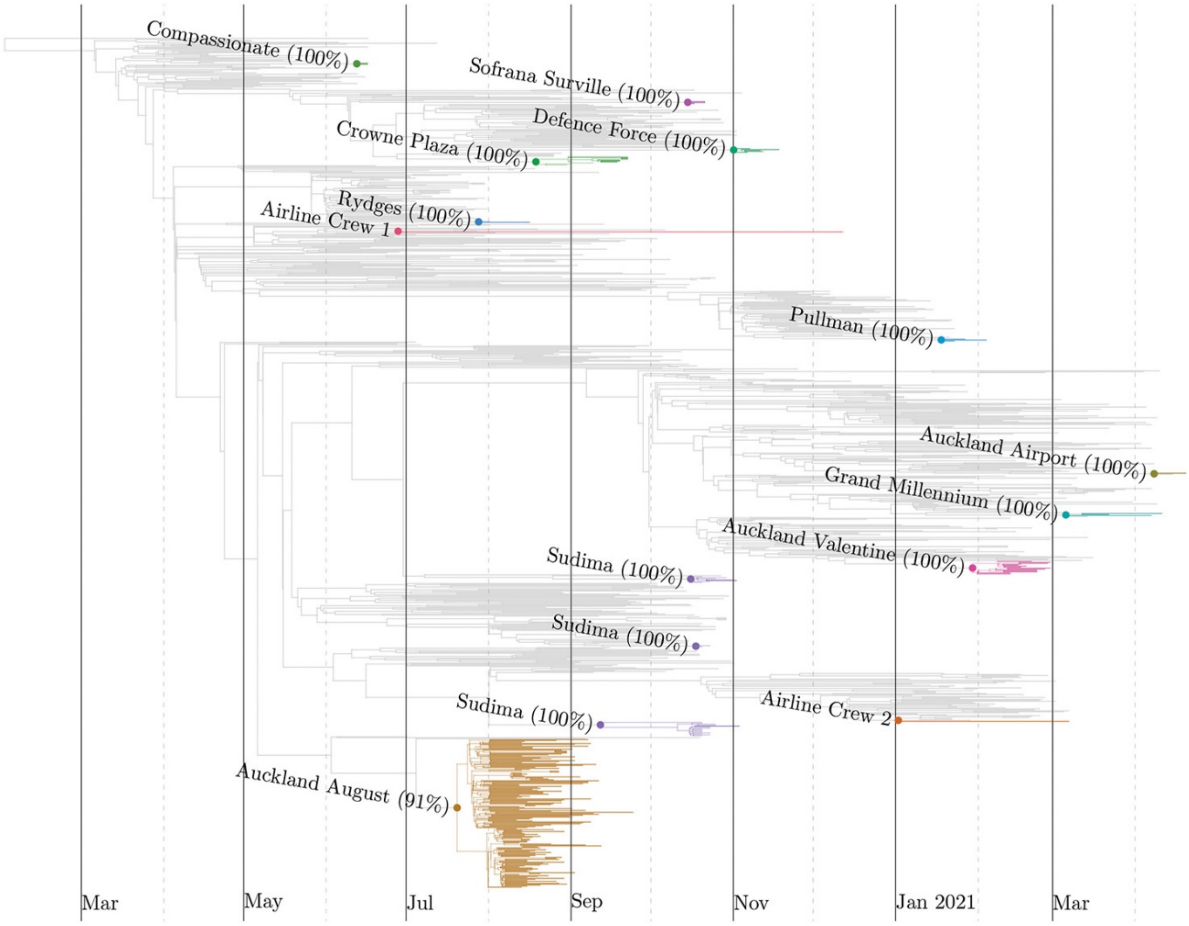

Appendix Figure. Complete phylogenetic tree for severe acute respiratory syndrome coronavirus 2. The 13 New Zealand community outbreaks are colored by cluster, and global genomic sequences are grey. The posterior clade supports (%) of each community outbreak are >90%, thus providing high confidence that each outbreak has a single origin (except for the Sudima outbreak, which has at least 3 origins). Note that the tree presented in Figure 1 of the main article was derived from this tree (by omitting global samples).
